# Assessment of the toxic effects of parabens, commonly used preservatives in cosmetics, and their halogenated by-products on human skin and endothelial cells

**DOI:** 10.1016/j.namjnl.2025.100011

**Published:** 2025-01-25

**Authors:** Alisha Janiga-MacNelly, Mackenna McGraw, Maria Teresa Fernandez-Luna, Ramon Lavado

**Affiliations:** a Department of Environmental Science, Baylor University, Waco, TX 76798, USA; b Department of Biology, Baylor University, Waco, TX 76798, USA

**Keywords:** Parabens, In vitro, Wound healing, Endothelial, Cytotoxicity

## Abstract

Parabens, widely used as preservatives in cosmetics, are increasingly concerning due to potential health risks, while their chlorinated and brominated by-products, found in aquatic environments, pose additional toxicity concerns. This study evaluated the toxic effects of parabens, their metabolite, and three halogenated by-products on human skin and endothelial cells using cytotoxicity and wound healing assays. Human epidermal keratinocytes (HEK001) and human dermal microvascular endothelial cells (HMEC-1) were used as models. In keratinocytes, butylparaben (BuP) and benzylparaben (BeP) were the most cytotoxic, with EC_50_ values of 1.52 ± 0.51 µM and 3.34 ± 0.97 µM, respectively. Halogenated parabens, such as methyl 3‑chloro-4-hydroxybenzoate (CMeP) and methyl 3,5-dibromo-4-hydroxybenzoate (DBMeP), also showed significant cytotoxicity, with EC_50_ values of 2.20 ± 0.76 µM and 1.49 ± 0.37 µM. Methylparaben (MeP), ethylparaben (EtP), and the metabolite 4-hydroxybenzoic acid (HBA) showed lower toxicity, with EC_50_ values ranging from 536 ± 178 µM to 1,313 ± 464 µM. In endothelial cells, MeP, EtP, and HBA had reduced toxicity, and halogenated by-products were less toxic, with EC_50_ values from 788 ± 140 µM to >10 mM. High concentrations (100 µM) of BuP, BeP, and halogenated by-products significantly inhibited wound healing in both cell types, while halogenated parabens inhibited keratinocyte proliferation at just 1 µM. This research enhances understanding of parabens' impact on wound healing, informing safety assessments for cosmetics and personal care products.

## Highlights

• Parabens and by-products pose cytotoxic risks to skin and endothelial cells.
• Butylparaben and benzylparaben showed high cytotoxicity in keratinocytes.
• Halogenated parabens inhibited keratinocyte proliferation at 1 µM.
• Parabens impaired wound healing at 100 µM concentrations.
• Findings inform cosmetic safety by highlighting paraben toxicity concerns.

## Graphical abstract

**Figure:**
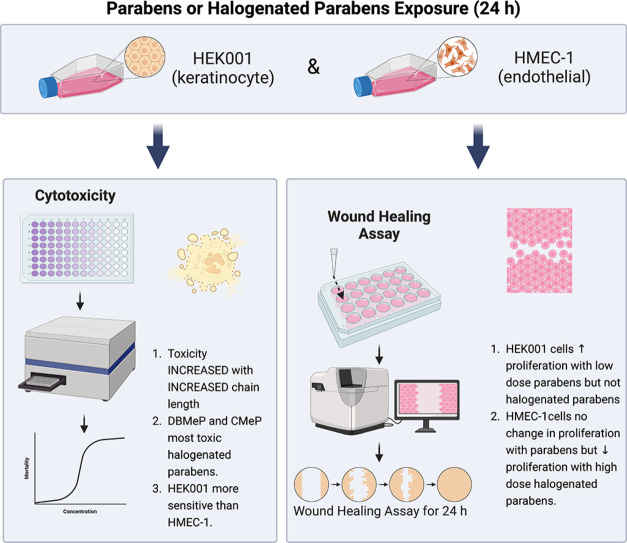

## 1 Introduction

Parabens are in the 4-hydroxybenzoate alky ester family and vary in chain length and degree of branching (Muktadir, 2023). They are colorless, odorless, and highly insoluble in water. However, the degree of solubility depends on the chain length (Chatterjee et al., 2024; Cherian et al., 2020; Muktadir, 2023). Parabens are commonly used as antimicrobials in cosmetics, pharmaceuticals, and personal care products, such as lipstick, makeup remover, powders, eyeshadow, lotions, soaps, shampoos, sunscreen, shaving gel, toothpaste, and mouthwash (Chatterjee et al., 2024; Resende et al., 2021; Wei et al., 2021). The degree of antimicrobial properties increases with paraben chain length but also depends on pH (Muktadir, 2023; Wei et al., 2021). Methylparaben (MeP), ethylparaben (EtP), propylparaben (PrP), butylparaben (BuP), and benzylparaben (BeP) are commonly used in cosmetic and personal care products at concentrations ranging from 100 to 3,200 μg/g in cosmetic products and up to 8,200 μg/g in personal care products (Wei et al., 2021).

In Europe, the European Union (EU) aligns with the Scientific Committee on Consumer Safety (SCCS) in their opinions on paraben safety. MeP and EtP are safe to use at concentrations <0.4 % (or 0.8 % in mixtures), but isoPrP, isoBuP, phenylparaben, BeP, and pentylparaben are banned. BuP and PrP can still be used in cosmetics at concentrations <0.19 % ((SCCS), 2013; Barroso, 2014). However, in the US, the Food and Drug Administration (FDA) doesn't regulate the use of parabens in cosmetics. Furthermore, FDA approval is not needed before putting paraben-based products on the market. As a result, parabens are ubiquitous in the environmental compartments and biological tissues. Urine samples from the US, Denmark, Sweden, China, Korea, Greece, and Spain tested positive for MeP, PrP, EtP, and BuP (Nowak et al., 2018). High concentrations (∼150 μg/L) of MeP and low levels of PrP were detected in the urine of pregnant women in France, Greece, Korea, Japan, Puerto Rico, and the US (Wei et al., 2021). Furthermore, parabens have been detected in indoor air systems, wastewater, and aquatic systems (Chatterjee et al., 2024; Dasmahapatra et al., 2024; Wei et al., 2021).

Acutely, parabens are believed to be nontoxic (Anderson, 2008; Cherian et al., 2020; Wei et al., 2021), but chronic exposure studies suggest they have carcinogenic, genotoxic, and endocrine disrupting properties (Cherian et al., 2020; Fransway et al., 2019; Wei et al., 2021). Toxicity increases with chain length largely due to increased hydrolysis time as seen in the crustacean *Daphnia magna,* the fish species *Pimephales promelas* (fathead minnows), *Oryzias latipes* (Japanese medaka), and green algae (Anderson, 2008; Bolujoko et al., 2021). *In vitro, in vivo,* and cadaver studies show dermal penetration increases with decreased chain length (Cherian et al., 2020; Fransway et al., 2019). Furthermore, damaged tissue favors the permeation of parabens through the skin (Resende et al., 2021). While rodent studies indicate that parabens are largely metabolized into 4-hydroxybenzoic acid (HBA), this process may be slower and less effective in human tissues ((SCCS), 2013; Darbre and Harvey, 2008).

Parabens gained mainstream media attention through their estrogenic and antiandrogenic properties. Animal studies show that paraben exposure can lead to abnormal reproductive organ weight and interfere with the function and development of male reproductive organs (Anderson, 2008; Chatterjee et al., 2024; Nowak et al., 2018). While the potency of parabens for estrogen receptors (ER) is low, there is a growing association between parabens and breast cancer (Cherian et al., 2020; Fransway et al., 2019). Prusakiewicz et al. (2007) found that parabens are likely estrogenic sulfotransferases (SULT) inhibitors, but a more recent investigation into this mechanism was not found.

While there is a vast amount of literature involving the endocrine disruption of parabens, there is very little available about how parabens may interact with wound healing processes. If a person has a cutaneous wound and is using a paraben-based dermal product, basal keratinocytes may come in contact with unmetabolized parabens. The skin has three layers: the epidermis, dermis, and hypodermis, which work to protect the body from UV radiation, chemical exposure, biological infection, and mechanical damage. The epidermis contains several layers, including *stratum corneum, stratum lucidum, stratum granulosum, stratum spinosum*, and *stratum basale*. In the *stratum basale* resides undifferentiated keratinocytes (basal keratinocytes), which continuously proliferate until they migrate and differentiate into epidermal keratinocytes. The dermis is host to the papillary and reticular layers, where blood vessels extend through to the hypodermis (Yousef et al., 2019).

When the skin is wounded, first, the body sends out an inflammatory response in which vessels constrict, and platelets and leukocytes are recruited. Following this, the proliferation of endothelial cells and angiogenesis begin to repair any damaged vessels (Gonzalez et al., 2016; Guo and DiPietro, 2010; Rodrigues et al., 2019). Basal keratinocytes increase proliferation and migration to reestablish the epithelial layer (Gonzalez et al., 2016; Rodrigues et al., 2019). Basal keratinocytes characteristically express keratin 14 and keratin 5, and this expression changes to keratin 10 and keratin 1 as they differentiate into epidermal keratinocytes (Suter et al., 2009). Some factors that influence the rate of wound healing include age, sex, diabetes, obesity, anti-inflammatory medications, and infection (Anderson and Hamm, 2012; Guo and DiPietro, 2010). While signal cascades to induce the proliferation of basal keratinocytes are highly complex and dependent on the response, it is well established that estrogen/estradiol, as well as activation of ERα/ERβ, increase keratinocyte proliferation (Merlo et al., 2009; Verdier-Sevrain et al., 2004; Zhou et al., 2016). If parabens are activating ER, it is expected that basal keratinocytes will increase proliferation. High concentrations of parabens have been shown to decrease cell viability, increase reactive oxygen species (ROS) production, induce apoptosis/necrosis, and damage DNA in keratinocytes (Chatterjee et al., 2024; Matwiejczuk et al., 2020; Nowak et al., 2018)). However, the effects of low concentration on keratinocyte proliferation are largely understudied.

This study employed a cytotoxicity assay to determine the concentrations of paraben derivatives that influence cellular viability and a proliferation assay to assess their effects on wound healing. To model basal keratinocytes, we selected the immortalized human epidermal keratinocyte cell line HEK001 due to its well-characterized features and relevance in dermatological and toxicological research. Derived from scalp tissue, HEK001 cells are notable for their high expression levels of keratin 10, which is crucial for epidermal differentiation; toll-like receptor (TLR) 5, involved in immune response modulation; and intercellular adhesion molecule-1 (ICAM-1) essential for inflammatory and reparative processes. These attributes make HEK001 cells a robust and reliable model for studying the mechanisms underlying wound healing and keratinocyte response to chemical exposure. This cell line has been extensively utilized in toxicological studies to evaluate key cellular functions, including wound healing, migration, and viability under various conditions. Its reproducible behavior and physiological relevance provide a valuable framework for elucidating the molecular and cellular pathways affected by paraben compounds. By employing HEK001 cells, this study offers insights into the specific responses of basal keratinocytes, which play a critical role in epidermal repair and homeostasis, while contributing to the broader understanding of the toxicological effects of indoles on skin health (Lisse and Rieger, 2017; Olaru and Jensen, 2010; Sugerman and Bigby, 2000).

In addition to re-epithelization, we were also interested in any effects parabens may have on angiogenesis. Estrogen is believed to protect against hypertension and apoptosis, as well as mediate NO production in endothelial cells (Chakrabarti et al., 2014; Rubanyi et al., 2002; Spyridopoulos et al., 1997). While parabens may be vasodilators, the toxic effects of parabens on endothelial cells are highly unknown (Anderson, 2008). In our study, we selected the immortalized human microvascular endothelial cell line (HMEC-1) as a representative model for capillary endothelial cells. This decision was guided by the distinct relevance of HMEC-1 cells to cutaneous wound healing research, as they more closely resemble the microvascular endothelial cells found in skin tissue compared to human umbilical vein endothelial cells (HUVEC), which are derived from large vessel endothelium. The microvascular endothelial environment plays a critical role in wound repair by facilitating angiogenesis, promoting cellular migration, and supporting tissue regeneration—processes that are not fully replicated by macrovascular models such as HUVEC. HMEC-1 cells are well-characterized as microvascular endothelial cells through their expression of key markers, including CD31 (PECAM-1), which is critical for endothelial cell-cell adhesion and migration; intercellular adhesion molecule-1 (ICAM-1), which mediates leukocyte adhesion and transmigration; and the endothelial-specific markers MoAB EN4 and PAL-E epitopes, which confirm their vascular origin and specialized function. These features underscore their physiological relevance for modeling capillary endothelial responses to various stimuli, including pro-healing factors and toxicological agents. Moreover, the HMEC-1 cell line offers several advantages, including reproducibility, stability in culture, and the ability to mimic the microvascular responses crucial for understanding wound healing dynamics. Their use in this study provides a robust and contextually appropriate platform to investigate the cellular and molecular mechanisms underpinning angiogenesis and endothelial response in cutaneous wound healing, thereby enhancing the translational significance of the findings (Ades et al., 1992).

To increase the novelty of our study, we included halogenated parabens in our analysis. When personal care products are washed off the body, they often come into contact with the chlorine and bromine ions used to treat water. Due to their kinetic favorability, the parabens' parent compounds rapidly undergo mono or di chlorination and/or bromination. The halogenated derivatives have proven to be highly resistant to oxidation reactions (Canosa et al., 2006). Swimming pools in Bejing tested positive for dichlorinated parabens at 5–11 ng/L (Li et al., 2015a) while river waters in Shizuoka City, Japan, contained 14–28 ng/L of dichloro-propylparaben (Terasaki et al., 2012). More recently, the Kitakami River tested positive for 10–110 ng/L of brominated parabens (Gouukon et al., 2020). Chlorinated and brominated parabens have also been detected in predatorial avian liver and kidney tissues, indicating possible biomagnification properties (Rojo et al., 2024). Studies utilizing animal and plant models have found that halogenated parabens increase oxidative stress, developmental stress, and decreased viability compared to the non-halogenated parent compounds. Furthermore, the di-halogenated compounds indicate greater toxicity than mono-halogenated parabens (de Souza Moura et al., 2024; dos Santos Gonçalves Nascimento et al., 2023; Yoon and Cho, 2024).

**Fig. 1:**
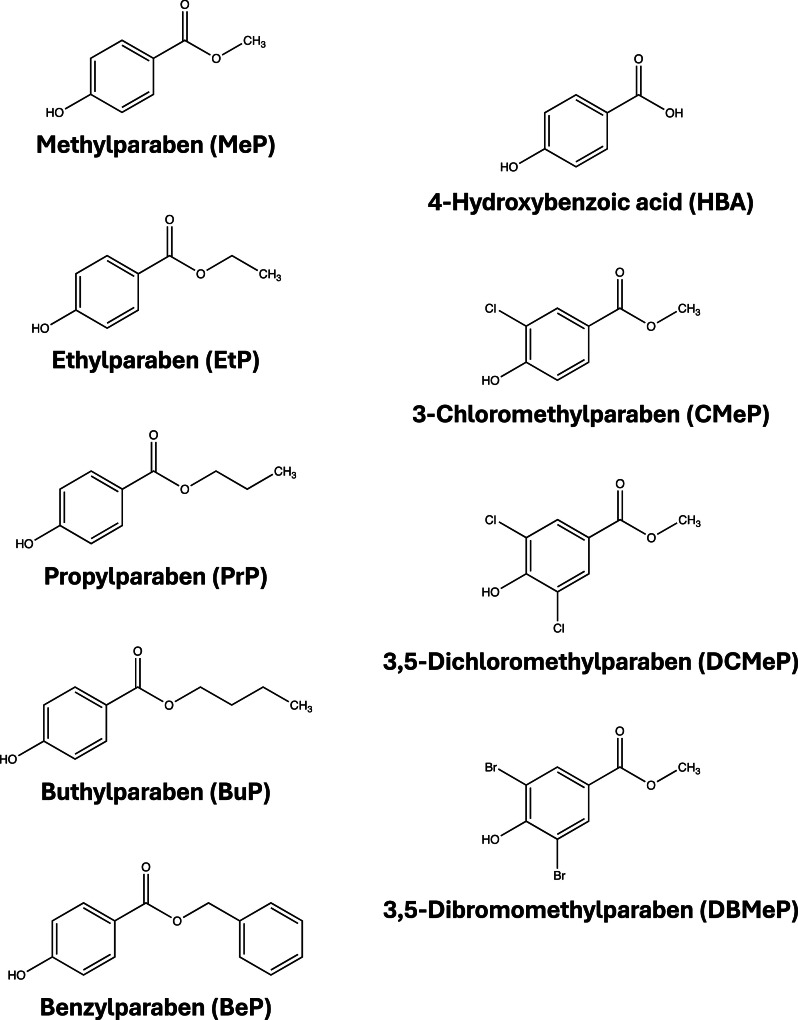
Chemical structures of the five parent parabens: methylparaben (MeP), ethylparaben (EtP), propylparaben (PrP), butylparaben (BuP), and benzylparaben (BeP), the primary metabolite 4-hydroxybenzoic acid (HBA); and three halogenated paraben by-products: methyl 3‑chloro-4-hydroxybenzoate (CMeP), methyl 3,5-dichloro-4-hydroxybenzoate (DCMeP), and methyl 3,5-dibromo-4-hydroxybenzoate (DBMeP) used in this study.

The goal of this investigation is to understand how parabens ranging in alkyl chain length and halogenation (five parental parabens, their metabolite, and three halogenated by-products) (Fig. 1) influence the cytotoxicity and proliferation of human basal keratinocytes and microvascular endothelial cells. We hope to address the knowledge gap of any potential effects parabens and halogenated parabens may have on human wound healing.

## 2 Material and methods

### 2.1 Chemicals and reagents

Keratinocyte serum-free media (SFM), human epidermal growth factor (EGF), MCDB-131 media, l-glutamine, penicillin G/streptomycin, phosphate-buffered saline (PBS), and trypsin–EDTA were obtained from Gibco Life Technologies (ThermoFisher Scientific, Waltham, MA). Hydrocortisone hemisuccinate, methyl 3‑chloro-4-hydroxybenzoate, and methyl 3,5-dichloro-4-hydroxybenzoate were obtained from Sigma-Aldrich (St. Louis, MO). Fetal bovine serum (FBS) was obtained from Atlas Biologicals (Fort Collins, CO). Deionized water (DI water) was obtained using a Milli-Q water purification system (Millipore, Billerica, MA). Methyl 4-hydroxybenzoate and dimethyl sulfoxide (DMSO) were purchased from Fisher Scientific (Waltham, MA). 4-hydroxybenzoic acid (4-HBA), ethyl, propyl, butyl, and benzyl 4-hydroxybenzoate (all ≥98 %) were purchased from TCI Chemicals (Tokyo, Japan). (3-(4,5-dimethylthiazol-2-yl)−2,5-diphenyltetrazolium bromide) (MTT) was obtained from Invitrogen (Carlsbad, CA).

### 2.2 Cell culture and dosing

The human epidermal keratinocytes HEK001 (CRL-2404, ATCC, Manassas, VA) were maintained in a keratinocyte-serum-free medium supplemented with 5 ng/mL human recombinant EGF. The cells were incubated under 5 % CO_2_ and a 95 % humidified atmosphere at 37 °C (Bogen et al., 2017). HMEC-1 cells (CRL-3243, ATCC) were cultured at 37 °C in a 5 % CO_2_ humidified atmosphere using MCDB-131 medium supplemented with 10 % fetal bovine serum, 1 % penicillin/streptomycin, 10 mM l-glutamine, 1 μg/mL hydrocortisone, and 10 ng/mL EGF supplement.

The experiments were performed with biological (96 well plates) and technical (wells) replicates. Four to five biological replicates with four technical replicates were used to determine cytotoxicity. Ten paraben concentrations were assessed, ranging from a low human serum-relevant concentration (1 pM) to a high non-human-relevant concentration (1 mM). A solvent control of 0.6 % DMSO and a positive control of 5 % DMSO were also used. Three representative concentrations were chosen for each cell line for the wound-healing assay. Those concentrations were 1 µM, 10 µM, and 100 µM. Upon dosing, plates were returned to the incubator for a 24-hour exposure period.

### 2.3 Cytotoxicity

Cell viability was assessed using the MTT assay [3-(4,5-dimethylthiazol-2-yl)−2,5-diphenyltetrazolium bromide] (Solan et al., 2022). After 24 h of exposure, a 20 % tetrazolium dye solution was added to the cells. The NAD(P)H-dependent oxidoreductase enzymes in viable cells reduced the tetrazolium dye, producing insoluble formazan crystals visible as a purple coloration, indicative of active cellular respiration. Following the incubation period, the media was carefully removed, leaving the formazan crystals behind. To dissolve these crystals, a 1:1 solution of ethanol and DMSO was added to the plate. Absorbance was then measured at 595 nm on a spectrophotometer to quantify the extent of formazan formation (BioTek SynergyTM H1, Winooski, VT).

A 24-hour exposure period was selected to evaluate the acute toxicity of parabens on HEK001 and HMEC-1 cell lines. While extensively studied cell lines such as HaCaT and HUVEC provide substantial prior data on the acute cytotoxic effects of parabens, limited or no comparable data exist for HEK001 and HMEC-1. Assessing the acute effects on these less-characterized cell lines is critical for enabling meaningful comparisons across models. Additionally, acute toxicity studies minimize confounding factors such as nutrient depletion and cell overgrowth, facilitating higher-throughput screening. Although 24-hour exposures do not account for long-term effects, they provide an essential foundation for toxicity assessment and generate preliminary evidence to justify subsequent investigations into chronic exposure scenarios.

### 2.4 Wound healing assay

As described by Grada et al. (2017) and Yarrow et al. (2004), a wound-healing assay was used to evaluate the proliferation rate of a disrupted cell monolayer. Cells were seeded at 200,000 cells/mL in 48-well plates and allowed to stabilize, reaching 90–100 % confluency within 24–72 h. Once confluent, the growth media was removed, and a scratch was created in the cell monolayer using a pipette tip. Manual disruption of the cell monolayer was chosen over the use of inserts or automated scratching devices to design experiments that are cost-effective and accessible to laboratories with limited budgets. This approach provides greater flexibility in experimental design without reliance on specialized equipment, making it a practical solution for resource-constrained settings. Additionally, the manual scratch method may better simulate real-world injury scenarios. To minimize variability, consistent pipette tip sizes were employed, experiments were performed in multiple replicates, and immediate visual inspections were conducted to ensure reproducibility and uniformity across trials. The exposure media was then applied, and cytochalasin D (1 mM) was used as a positive control to inhibit wound healing by suppressing cell division via microtubule action. Cells were incubated with exposure media for 24 h while kinetic high contrast brightfield (HCBF) images were captured every 30 min using the Lionheart FX automated microscope (Biotek, Agilent). Gen5 software analyzed the scratch area over time, measuring the reduction in cell-free area as migration occurred. All data were normalized to the solvent control for consistency.

### 2.5 Data analysis

Our data was analyzed before statistical analysis to meet the homoscedasticity and normality assumptions of parametric tests. Statistical significance was assessed using one-way ANOVA tests to evaluate differences between concentrations and EC_50_ s, with the use of GraphPad Prism version 10.00 for MacOS (GraphPad Software, San Diego, CA, USA). A *p*-value of <0.05 was considered statistically significant unless otherwise indicated (Lavado et al., 2014). If an overall significance was detected, Dunnett's multiple range tests were performed to compare the toxic effects of parabens.

## 3 Results

### 3.1 Cytotoxicity

**Fig. 2:**
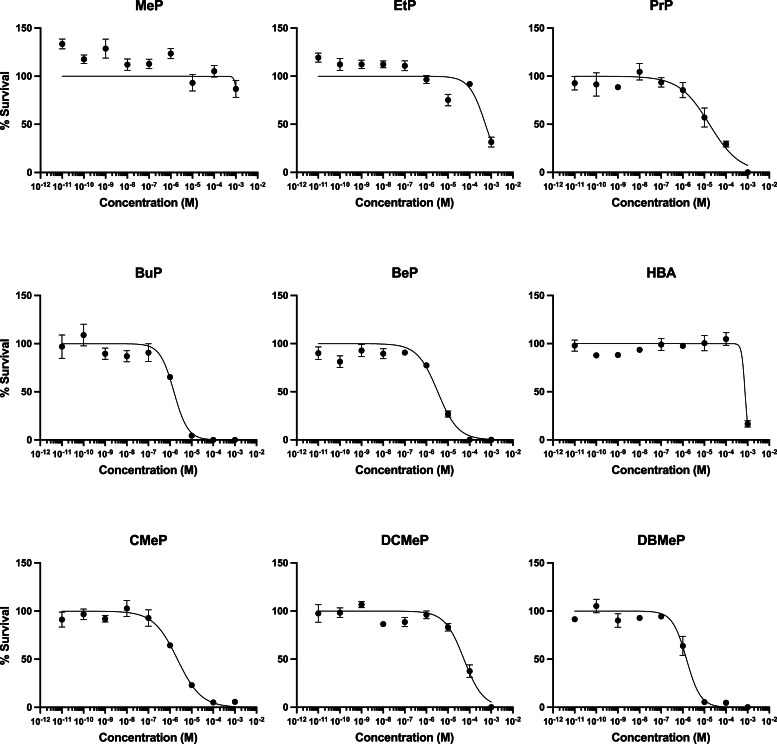
Dose-response curves for cytotoxic evaluation (EC_50_) in M following 24 h exposures of HEK001 cells (keratinocytes) to methylparaben (MeP), ethylparaben (EtP), propylparaben (PrP), butylparaben (BuP), benzylparaben (BeP), 4-hydroxybenzoic acid (HBA), chloromethylparaben (CMeP), dichloromethylparaben (DCMeP), and dibromomethylparaben (DBMeP). Data are presented as average ± standard deviation in µM (4–5 biological replicates with 4–8 technical replicates).

In HEK001 cells, BuP was the most toxic paraben (EC₅₀ = 1.52 ± 0.51 µM), followed by BeP (EC₅₀ = 3.34 ± 0.97 µM). Among other paraben compounds, toxicity increased with chain length in the order of PrP > EtP > MeP. The halogenated parabens, DBMeP (EC₅₀ = 1.5 ± 0.4 µM) and CMeP (EC₅₀ = 2.2 ± 0.8 µM) were significantly more toxic than DCMeP (EC₅₀ = 54.5 ± 17.84 µM). The metabolite HBA exhibited low toxicity, with an EC₅₀ of 696.2 ± 244.7 µM (Table 1 and Fig. 2).

**Table 1:** EC_50_ s produced from cytotoxicity assay for the human cell lines, HEK001 (keratinocytes) and HMEC-1 (endothelial) exposed to methylparaben (MeP), ethylparaben (EtP), propylparaben (PrP), butylparaben (BuP), benzylparaben (BeP), 4-hydroxybenzoic acid (HBA), chloromethylparaben (CMeP), dichloromethylparaben (DCMeP), and dibromomethylparaben (DBMeP). Data are presented as average ± standard deviation in µM (4–5 biological replicates with 4–8 technical replicates).

	Cell Line	
	HEK001	HMEC-1
**Parental**		
** MeP**	1313.0 ± 464.2	1087.0 ± 618.4
** EtP**	536.5 ± 177.9	171.2 ± 77.9
** PrP**	17.0 ± 9.9	101.9 ± 18.0
** BuP**	1.5 ± 0.51	75.8 ± 13.4
** BeP**	3.3 ± 1.0	25.64 ± 3.9
**Metabolite**		
** HBA**	692.2 ± 244.7	> 10,000
**Halogenated**		
** CMeP**	2.2 ± 0.8	788.0 ± 139.3
** DCMeP**	54.5 ± 17.84	> 10,000
** DBMeP**	1.5 ± 0.4	488.8 ± 86.41

**Fig. 3:**
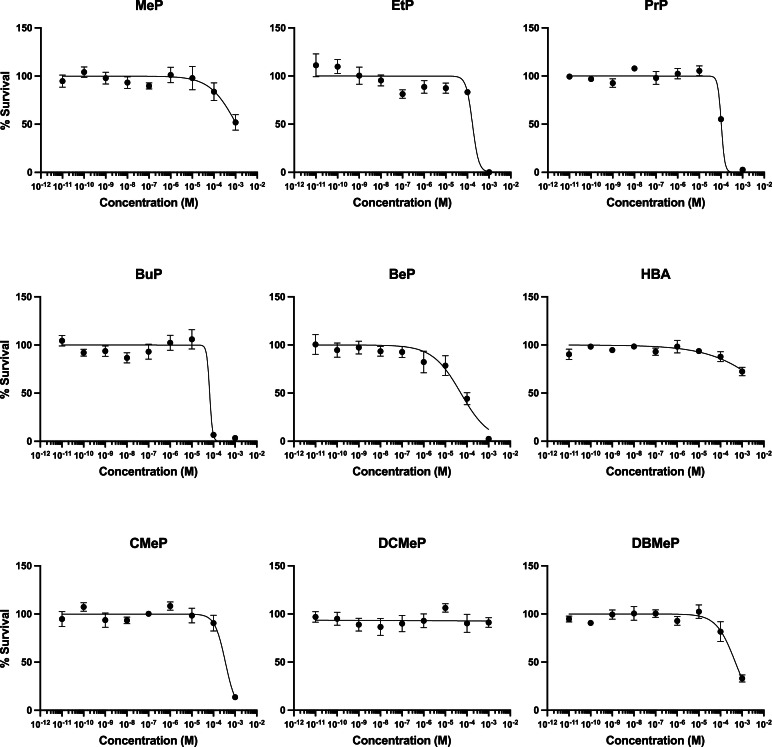
Dose-response curves for cytotoxic evaluation (EC_50_) in M following 24 h exposures of HMEC-1 cells (endothelial) to methylparaben (MeP), ethylparaben (EtP), propylparaben (PrP), butylparaben (BuP), benzylparaben (BeP), 4-hydroxybenzoic acid (HBA), chloromethylparaben (CMeP), dichloromethylparaben (DCMeP), and dibromomethylparaben (DBMeP). Data are presented as average ± standard deviation in µM (4–5 biological replicates with 4–8 technical replicates).

Similar trends in chain length and halogenation were observed in HMEC-1 cells, though the impact on cell viability was less pronounced. BuP (EC₅₀ = 75.8 ± 13.4 µM) and BeP (EC₅₀ = 25.64 ± 3.9 µM) remained the most toxic, with PrP > EtP > MeP. However, EC₅₀ values in HMEC-1 cells were significantly higher than those in HEK001 cells for all exposures except MeP. This pattern was also observed for HBA and the halogenated parabens (Table 1 and Fig. 3).

### 3.2 Wound healing assay

**Fig. 4:**
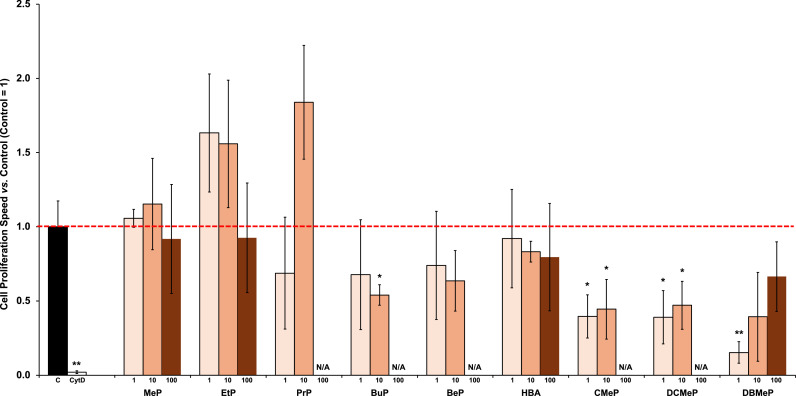
Wound-healing assay observed in HEK001 cells exposed to methylparaben (MeP), ethylparaben (EtP), propylparaben (PrP), butylparaben (BuP), benzylparaben (BeP), 4-hydroxybenzoic acid (HBA), chloromethylparaben (CMeP), dichloromethylparaben (DCMeP), and dibromomethylparaben (DBMeP) for 24 h. Data are presented as average ± standard deviation and as cell proliferation speed normalized to solvent-exposed cells (**C**, Control). **CytD** represents the positive control as cells exposed to the well-characterized cell proliferation inhibitor cytochalasin D. **N/A**: Not determined due to high cytotoxicity levels. Asterisks represent treatments with significant differences (* means *p* < 0.05; ** means *p* < 0.01) relative to the solvent control (One-way ANOVA, Dunnett's test; 4–5 biological replicates with 4–8 technical replicates).

The goal of the wound healing assay was to test a low, medium, and high dose representing a broad spectrum of concentrations. However, based on the results of the cytotoxicity assay, high concentrations (100 µM) of PrP, BuP, BeP, and chlorinated by-products acutely lethal responses in HEK001 cells. As a result, proliferation could not be observed in these compounds at 100 µM in this cell line. In MeP and EtP exposures of 100 µM, there is a substantial decrease in proliferation rates. Conversely, at 100 µM DBMeP, there is a dose-dependent increase in proliferation. There is also an increase in proliferation with 10 µM of PrP, EtP, and MeP and at 1 µM of BuP and BeP. However, at 10 µM of BuP and BeP, proliferation significantly decreased (*p* < 0.05, One-way ANOVA, Dunnett's test). All of the halogenated by-products produced significantly lower rates of proliferation (∼40 % of the control, *p* < 0.05, One-way ANOVA, Dunnett's test) compared to the non-halogenated parent compound (Fig. 4).

**Fig. 5:**
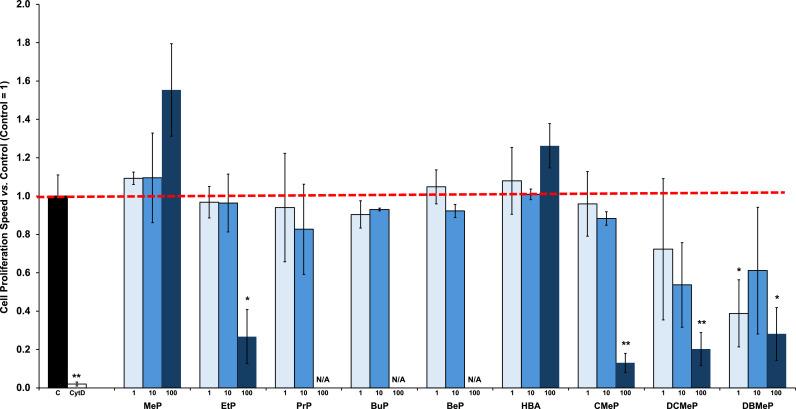
Wound-healing assay observed in HMEC-1 cells exposed to methylparaben (MeP), ethylparaben (EtP), propylparaben (PrP), butylparaben (BuP), benzylparaben (BeP), 4-hydroxybenzoic acid (HBA), chloromethylparaben (CMeP), dichloromethylparaben (DCMeP), and dibromomethylparaben (DBMeP) for 24 h. Data are presented as average ± standard deviation and as cell proliferation speed normalized to solvent-exposed cells (**C**, Control). **CytD** represents the positive control as cells exposed to the well-characterized cell proliferation inhibitor cytochalasin D. **N/A**: Not determined due to high cytotoxicity levels. Asterisks represent treatments with significant differences (* means *p* < 0.05; ** means *p* < 0.01) relative to the solvent control (One-way ANOVA, Dunnett's test; 4–5 biological replicates with 4–8 technical replicates).

Similar to HEK001 cells, HMEC-1 cells were too sensitive to conduct proliferation assays at 100 µM for PrP, BuP, and BeP. However, exposures to 1–10 µM of MeP, EtP, PrP, BuP, BeP, and CMeP resulted in proliferation rates comparable to those observed in untreated controls. At 100 µM, EtP, CMeP, DCMeP, and DBMeP significantly reduced proliferation rates in HMEC-1 cells to 20–30 % of the baseline. In contrast, 100 µM of MeP caused a significant increase in proliferation rates, reaching 160 % of the untreated control levels (Fig. 5).

## 4 Discussion

In this study, cytotoxicity evaluations revealed that MeP and HBA exhibited the least cytotoxic effects across both cell lines. In contrast, DBMeP and BuP significantly decreased cell viability, with the lowest EC_50_ values, followed by CMeP>BeP>PrP>DCMeP>EtP>HBA>MeP. These results suggested a strong relationship between paraben chain length and toxicity, with di-bromination being more cytotoxic to HEK001 cells than dichlorination. A similar relationship between paraben chain length and cytotoxicity has also been observed in other dermal cell lines, such as KERTr, HaCaT (Chrz et al., 2020), and HDFn (Kizhedath et al., 2019), as well as in the liver carcinoma cell line HepG2 (Khanal et al., 2012).

There is strong evidence that parabens induce apoptosis through caspase pathways. For example, in M624 melanoma cells and HaCaT keratinocytes, exposure to 200 µM of heptylparaben significantly reduced cell viability, increased cytochrome C levels, and activated caspase 3 (Couch, 2024; Kinney, 2022). Similarly, in HepG2 cells, 125 µM of BuP led to a higher percentage of apoptotic cells, increased expression of pro-apoptotic proteins Bax and caspase 3, and decreased levels of the anti-apoptotic protein Bcl2 compared to HBA, MeP, and PrP (Khanal et al., 2012). Additionally, BuP has been shown to cause cell cycle arrest in the G1 phase in both HepG2 and HDFn cells at the same concentration (Kizhedath et al., 2019). While these concentrations are higher than typical human exposure levels, they are important for understanding the mechanisms of paraben toxicity. While apoptosis was not studied in this investigation, the reduced cell viability from the cytotoxicity assay supports the claim that parabens can be toxic to *in vitro* cell lines. Further research into the mechanism of action between paraben compounds and basal keratinocytes is needed to confirm this hypothesis.

In this study, the HMEC-1 endothelial cell line demonstrated similar patterns of toxicity when compared to the HEK001 keratinocyte cell line. However, the EC_50_ values in HMEC-1 cells were 1–2 orders of magnitude higher than those observed in HEK001 cells, indicating that HMEC-1 cells exhibit lower sensitivity to parabens. While there are a few studies on the effects of paraben effects on red blood cells (RBCs) and vasodilation, the current literature on endothelial paraben toxicity is notably sparse. This study represents the first known investigation utilizing HMEC-1 cells as a model for evaluating paraben toxicity. Ko et al. (2023) investigated the effects of MeP, EtP, PrP, BuP, and HBA on RBCs and found that only BuP exposure was linked to an increase in extracellular phosphatidylserine. When RBCs were exposed to 500 µM of BuP, they exhibited increased hemolysis, microvesicle generation, intracellular calcium levels, scramblase activity, thrombin generation, and a reduction in flippase activity. The study also highlighted that these effects were intensified under conditions of elevated shear stress, which is commonly observed in patients with hypertension. In a related human study conducted in China, exposure to EtP and PrP was associated with increased risks of hypertension and high blood pressure. The median urinary concentrations of MeP, EtP, PrP, and BuP were reported as 35.45 µg/L, 1.13 µg/L, 1.19 µg/L, and 0.44 µg/L, respectively (Zhang et al., 2023). The acute cytotoxic effects of paraben exposure were markedly lower in HMEC-1 cells compared to HEK001 cells; however, this trend may differ under chronic exposure conditions. This underscores the necessity of further research to evaluate the cytostatic and chronic toxicity effects of parabens on endothelial cells.

Despite the paucity of research on the impact of parabens on endothelial cells, it is well-known that parabens are weakly estrogenic, and estrogenic activity increases with chain length (Anderson, 2008; Fransway et al., 2019; Muktadir, 2023). In keratinocytes, estrogen promotes proliferation, which in turn supports wound healing. This is likely mediated through interactions with TGF-β1, Erk, Akt, and PCNA pathways (Merlo et al., 2009; Zhou et al., 2016). Conversely, in endothelial cells, estrogen plays a crucial role in promoting vasodilation, activating GPR30 signaling cascades, and increasing nitric oxide (NO) bioavailability (Chakrabarti et al., 2014; Rubanyi et al., 2002). Estrogen is believed to protect against hypertension and promotes angiogenesis (Arnal et al., 2010; Rubanyi et al., 2002). However, the involvement of hematopoietic cells may modulate the angiogenic response (Arnal et al., 2010).

In this study, HEK001 cells exposed to BuP and BeP at concentrations of 1 µM and 10 µM exhibited lower proliferation rates compared to the solvent control (Fig. 4), although the differences were not statistically significant. It was anticipated that proliferation would be absent at the 10 µM concentration due to its cytotoxic effects. However, proliferation rates at this concentration were comparable to those of the solvent control. These discrepancies may arise from the limitations in the sensitivity of the experimental methods employed. For instance, cells may possess mechanisms that enable them to recover or adapt to low-level toxic insults over short durations, thereby temporarily maintaining proliferation despite exposure to cytotoxic concentrations. Alternatively, the cytotoxic effects of BuP and BeP at 10 µM might be near a threshold where they are insufficient to fully inhibit proliferation, and slight variability in cellular responses could result in some degree of proliferation. Furthermore, while 10 µM BuP and BeP could be toxic to a subset of cells, other cells may remain viable and continue to proliferate, leading to overall rates similar to the control. Nonlinear dose-response effects may also play a role, wherein intermediate concentrations elicit distinct cellular behaviors compared to lower or higher concentrations. Another possibility is that BuP and BeP induce cell cycle arrest rather than outright cytotoxicity, temporarily halting proliferation without complete cell death. Further research, particularly on wound healing and proliferation across a broader range of concentrations between 1 µM and 10 µM, is essential to resolve these ambiguities and provide a clearer understanding of the observed cellular responses.

In contrast, in HMEC-1 endothelial cells, exposure to the same parabens at concentrations resulted in proliferation rates comparable to the solvent control (Fig. 5). This differential response may be attributed to the more prominent role of estrogen in regulating cell proliferation and the cell cycle in keratinocytes compared to endothelial cells. As HEK001 cells are potentially more responsive to the estrogenic activity of parabens, they may be more prone to estrogen-induced apoptotic pathways, which could explain the lower EC_50_ values observed in HEK001 cells relative to HMEC-1 cells. However, further investigation is required to confirm this hypothesis, including a comparative analysis of paraben toxicity, estrogenic activity, and cell cycle progression between keratinocytes and endothelial cells.

Parabens in the environment are highly susceptible to halogenation, often forming chlorinated and brominated derivatives. For instance, dichlorinated parabens made up 44 % of the total parabens detected in water samples from pools and rivers in Japan (Terasaki et al., 2015b). Similarly, a variety of halogenated parabens were found in swimming pools in Beijing, China, including DCEtP (11.1 ng/L), DCMeP (4.87 ng/L), CEtP (0.02 ng/L), and CMeP (1.49 ng/L) (Li et al., 2015b). Brominated parabens have also been detected in tap water, with average concentrations of 12.8 ng/L (Albouy et al., 2023). Moreover, CMeP, CEtP, DCMeP, and DBEtP have been identified in birds of prey (Rojo et al., 2024). Notably, halogenated parabens exhibit stronger endocrine-disrupting effects compared to their parent compounds (Jakopin, 2021). While these studies provide valuable insights into halogenated parabens, significant gaps remain in our understanding of the toxicity of chlorinated and brominated parabens.

Our study demonstrated that the cytotoxicity of halogenated parabens in both HEK001 and HMEC-1 cell lines followed the hierarchy: DBMeP > CMeP > DCMeP. HEK001 cells were significantly more sensitive to these compounds than HMEC-1 cells. Similarly, *in vitro* studies on the human liver (HepaRG), human colorectal (Caco-2), fish liver (RTL-W1, PLHC-1), and fish gill (GB1, RTgill-W1) consistently showed that DBMeP exhibited higher cytotoxicity compared to DCMeP and CMeP. Interestingly, DCMeP was more toxic than CMeP in liver and gill cells but not in Caco-2 cells (Ball et al., 2023). In *Daphnia*, DCMeP exhibited greater toxicity than CMeP, potentially causing nonspecific membrane disruption (Terasaki et al., 2009). Medaka fish were most sensitive to halogenated MeP, with toxicity following the order: DBMeP > DCMeP > CMeP (Yoon and Cho, 2024). These findings highlight DBMeP's higher toxicity relative to chlorinated parabens, although species- and cell-line-specific differences in chlorinated paraben toxicity are evident. Further comparative analysis across species and degrees of chlorination is needed to better understand the toxicological impacts of halogenated parabens.

In HEK001 cells, exposure to CMeP and DCMeP at concentrations of 1–10 µM led to a decrease in proliferation and significant cell death at 100 µM. In contrast, HMEC-1 cells showed no significant changes in proliferation at 1–10 µM, though a reduction was noted at 100 µM. Interestingly, DBMeP exposure in HEK001 cells initially reduced proliferation at 1 µM, but at higher concentrations, a dose-dependent increase in proliferation was observed, suggesting that DBMeP may operate through a different mechanism than the chlorinated parabens. In HMEC-1 cells, DBMeP also caused a reduction in proliferation, though trends were less consistent due to high variability. These results suggest that while CMeP and DBMeP exhibit greater cytotoxicity than DCMeP, and DBMeP may engage distinct metabolic pathways.

In yeast models, monochlorinated parabens were found to be significantly more potent activators of aryl hydrocarbon receptors (AhR) compared to dichlorinated derivatives, with a potency comparable to aza-PAHs (Terasaki et al., 2015a). Additionally, brominated and dibrominated parabens displayed strong estrogen antagonistic effects, unlike their non-halogenated parent compounds (Saski and Terasaki, 2018). This estrogen antagonism may explain the observed decrease in proliferation at lower doses of halogenated parabens in HEK001 cells but not in HMEC-1 cells. These findings suggest that the cytotoxicity and endocrine-disrupting effects of halogenated parabens may vary depending on the halogenation pattern and target cell type, with DBMeP potentially acting through unique receptor pathways compared to chlorinated derivatives. However, additional research into AhR potency and ER activity is needed to better establish these relationships.

Several models indicate that halogenated parabens are stronger inducers of oxidative stress than their parent compounds. Plant studies indicate that chlorinated paraben derivatives lead to greater reductions in root growth, cell proliferation impairments, and decreased cell viability compared to parent compounds (dos Santos Gonçalves Nascimento et al., 2023). Furthermore, chlorinated parabens significantly decreased CAT activity, increased GPX activity, and amplified lipid peroxidation (de Souza Moura et al., 2024; dos Santos Gonçalves Nascimento et al., 2023). In worms, DCMeP led to increased CAT, APX, and GPX activity, and worms were more likely to evacuate contaminated soil than with non-halogenated exposure (dos Santos Gonçalves Nascimento et al., 2023). Halogenated parabens were more potent inducers of ROS, CAT activation and were associated with decreased hatching success, reduced heart rate, and elevated deformities in medaka embryos (Yoon and Cho, 2024). Collectively, these results suggest that halogenated and non-halogenated parabens may have different pathways of toxicity. Specifically, halogenated parabens are strong activators of oxidative stress pathways, while non-halogenated parabens tend to activate apoptosis pathways. However, a deeper analysis of the oxidative stress impacts in mammalian and human models is needed to better establish this conclusion. There may be similarities in the mechanisms of action of these two types of parabens that were not identified in this study, and potential interspecies differences in paraben toxicity may exist; however, more detailed inquiries are required to fully disclose these patterns and their implications for toxicity.

## 5 Conclusions

The experimental evidence indicates that increased molecular branching enhances toxicity, with halogenated parabens generally exhibiting greater toxicity than their non-halogenated counterparts. Consistently, HEK001 cells demonstrated higher sensitivity to paraben toxicity compared to HMEC-1 cells. The differential role of estrogen in cellular processes between keratinocytes and endothelial cells may explain the heightened sensitivity of HEK001 cells to estrogenic parabens compared to HMEC-1 cells. This observed variability in cell line responses underscores the importance of considering cell type- and species-specific reactions when evaluating paraben toxicity. Halogenated parabens such as DBMeP, CMeP, and DCMeP display increased cytotoxicity and distinct mechanisms of action compared to non-halogenated parabens. In HEK001 cells, halogenated parabens led to reduced proliferation, a pattern not observed in HMEC-1 cells, suggesting possible estrogen antagonism. The mechanistic divergence between halogenated and non-halogenated parabens likely contributes to differences in adverse biological outcomes. These findings emphasize the need for further research to elucidate the specific mechanisms driving halogenated paraben toxicity, particularly across different species, cell types, and halogenation patterns, to better understand their potential ecological and health impacts.

## CRediT authorship contribution statement

**Alisha Janiga-MacNelly:** Writing – original draft, Visualization, Validation, Methodology, Investigation, Formal analysis. **Mackenna McGraw:** Writing – original draft, Visualization, Validation, Methodology, Investigation, Formal analysis, Conceptualization. **Maria Teresa Fernandez-Luna:** Writing – review & editing, Resources, Project administration, Methodology, Investigation, Formal analysis. **Ramon Lavado:** Writing – review & editing, Visualization, Validation, Supervision, Project administration, Funding acquisition, Formal analysis, Data curation, Conceptualization.

## Declaration of interests

The authors declare that they have no known competing financial interests or personal relationships that could have appeared to influence the work reported in this paper.

## Data Availability

Data will be made available on request.
